# Adenomatoid mesothelioma arising from the diaphragm: a case report and review of the literature

**DOI:** 10.1186/s13256-022-03420-9

**Published:** 2022-05-30

**Authors:** Kenta Kawabe, Hiroki Sato, Akiko Kitano, Ryuichi Yoshida, Kazuya Yasui, Yuzo Umeda, Kazuhiro Yoshida, Tomokazu Fuji, Kenjiro Kumano, Kosei Takagi, Masaaki Kagoura, Takahito Yagi, Toshiyoshi Fujiwara

**Affiliations:** 1 Center for Graduate Medical Education, Okayama University Hospital, 2-5-1 Shikata-cho, Kita-ku, Okayama, 700-8558 Japan; 2 Department of Gastroenterological Surgery, Okayama University Hospital, 2-5-1 Shikata-cho, Kita-ku, Okayama, 700-8558 Japan; 3 Department of Pathology, Okayama University Hospital, 2-5-1 Shikata-cho, Kita-ku, Okayama, 700-8558 Japan

**Keywords:** Adenomatoid mesothelioma, Adenomatoid tumor, Mesothelial tumor, Diaphragm, Peritoneal

## Abstract

**Background:**

Adenomatoid mesothelioma is a rare subtype of malignant mesothelioma that can be confused with adenomatoid tumors, which are classified as benign. The clinical features and optimal management of adenomatoid mesothelioma have not been elucidated in the literature. In this report, we present an extremely rare case of adenomatoid mesothelioma that developed on the peritoneal surface of the diaphragm as well as a literature review of adenomatoid mesothelioma in the abdominal cavity.

**Case presentation:**

The patient was a 61-year-old Japanese woman who had undergone resection of a malignant peripheral nerve sheath tumor of the hand 18 years prior. She was diagnosed with clinical stage I lung adenocarcinoma on follow-up chest radiography. Simultaneously, a 20-mm enhancing nodule with slow growth on the right diaphragm was detected on contrast-enhanced computed tomography. She presented no specific clinical symptoms. At this point, the lesion was suspected to be a hypervascular tumor of borderline malignancy, such as a solitary fibrous tumor. After a left upper lobectomy for lung adenocarcinoma, she was referred to our department, and laparoscopic tumor resection was performed. Adenomatoid tumors were also considered based on the histopathological and immunohistochemical analyses, but we made the final diagnosis of adenomatoid mesothelioma using the results of the genetic profile. The patient remains alive, with no recurrence noted 6 months after surgery.

**Conclusion:**

We encountered a valuable case of adenomatoid mesothelioma of peritoneal origin. There are some previously reported cases of adenomatoid mesothelioma and adenomatoid tumors that may need to be recategorized according to the current classification. It is important to accumulate and share new findings to clarify the clinicopathological characteristics and genetic status of adenomatoid mesothelioma.

## Background

Malignant mesothelioma is classified into three main histological subtypes as follows: epithelial, sarcomatoid, and biphasic. According to the recent World Health Organization classification of pleural tumors, adenomatoid mesothelioma is categorized as a rare subtype of epithelioid mesothelioma that should be distinguished from adenomatoid tumors, which are benign mesothelial tumors [1, 2]. It is reported that adenomatoid mesothelioma accounts for approximately 5% of pleural mesothelioma and can present various types of morphologic features, leading to difficulty in diagnosis [3]. Due to the rarity of reported cases, the clinical features and optimal management of adenomatoid mesothelioma have not been described yet. Herein, we report an extremely rare case of adenomatoid mesothelioma that developed on the peritoneal surface of the diaphragm as well as a literature review of adenomatoid mesothelioma in the abdominal cavity.

## Case presentation

**Fig. 1:**
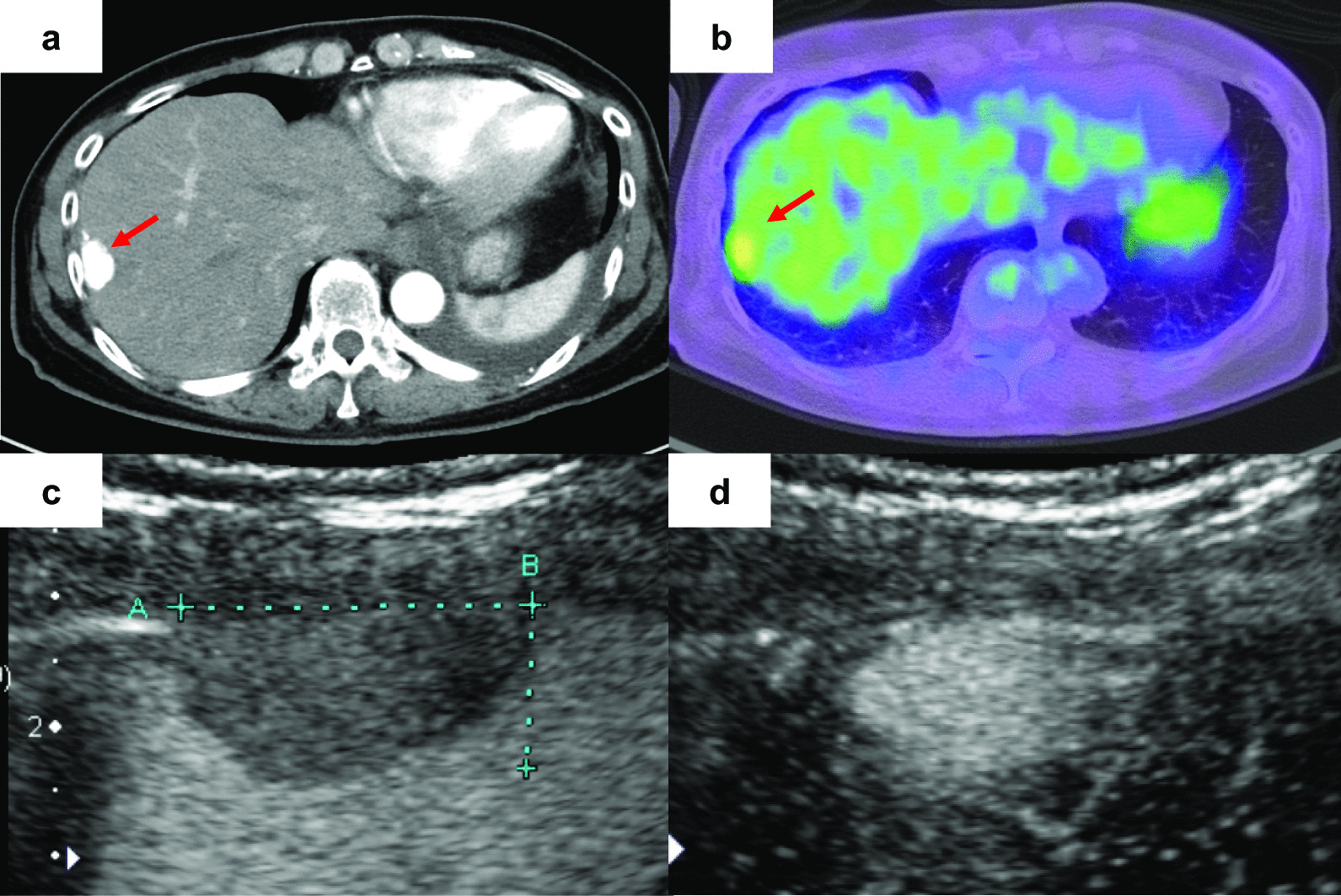
Preoperative examination.** a** Contrast-enhanced computed tomography revealed a tumor (arrow) with marked enhancement in the arterial phase of the right diaphragm.** b** 18-Fluoro-2-deoxyglucose positron emission tomography image revealed that the maximal standard uptake value of nodule (arrow) was 3.5.** c** Ultrasonography showed a low-echoic lesion.** d** Early enhancement was observed in Sonazoid-enhanced ultrasonography

**Fig. 2:**
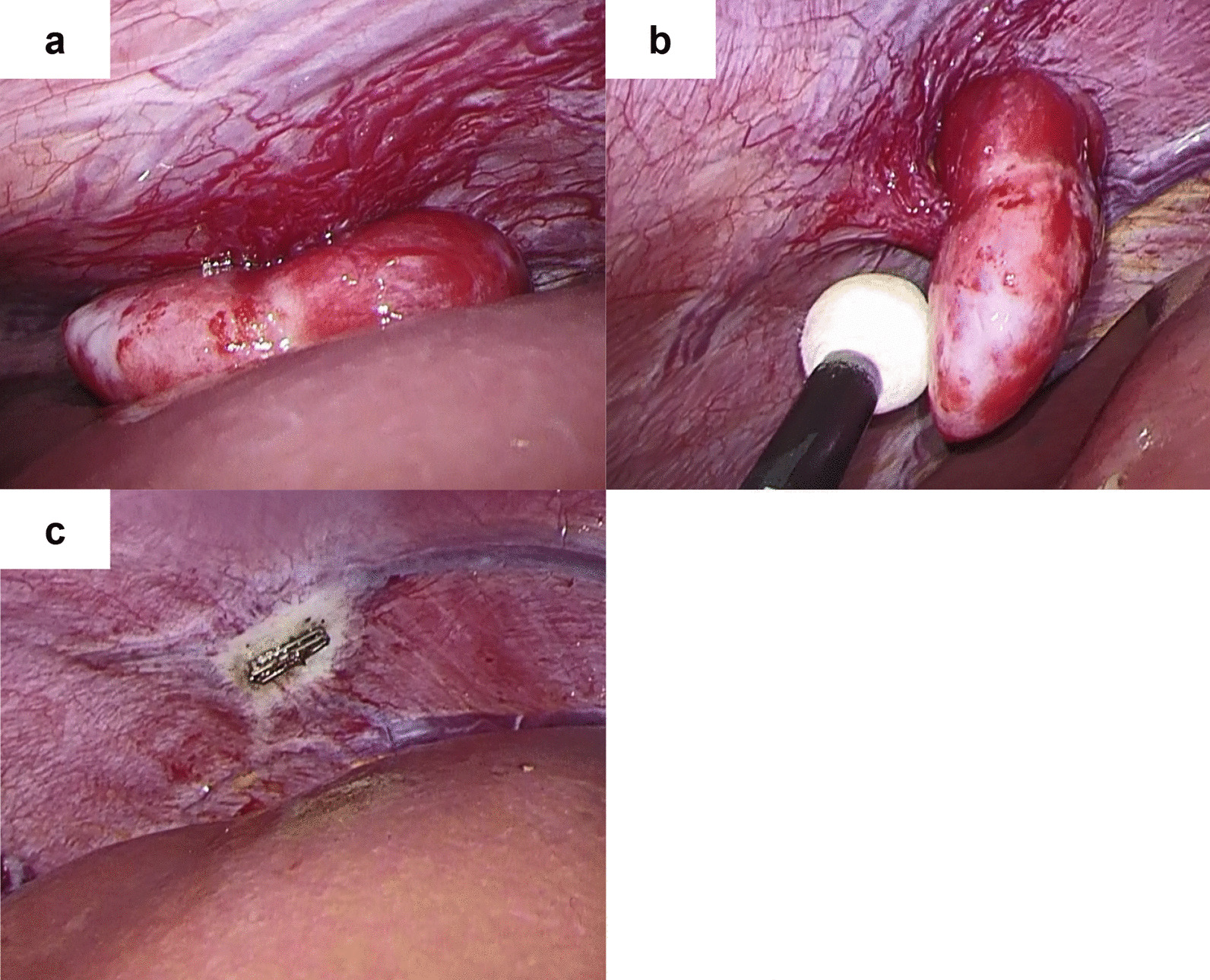
Intraoperative findings. **a** A pedunculated tumor with a thin pedicle originating from the peritoneal surface of the right diaphragm. **b** The pedicle of the tumor was clipped, and the tumor was excised

**Fig. 3:**
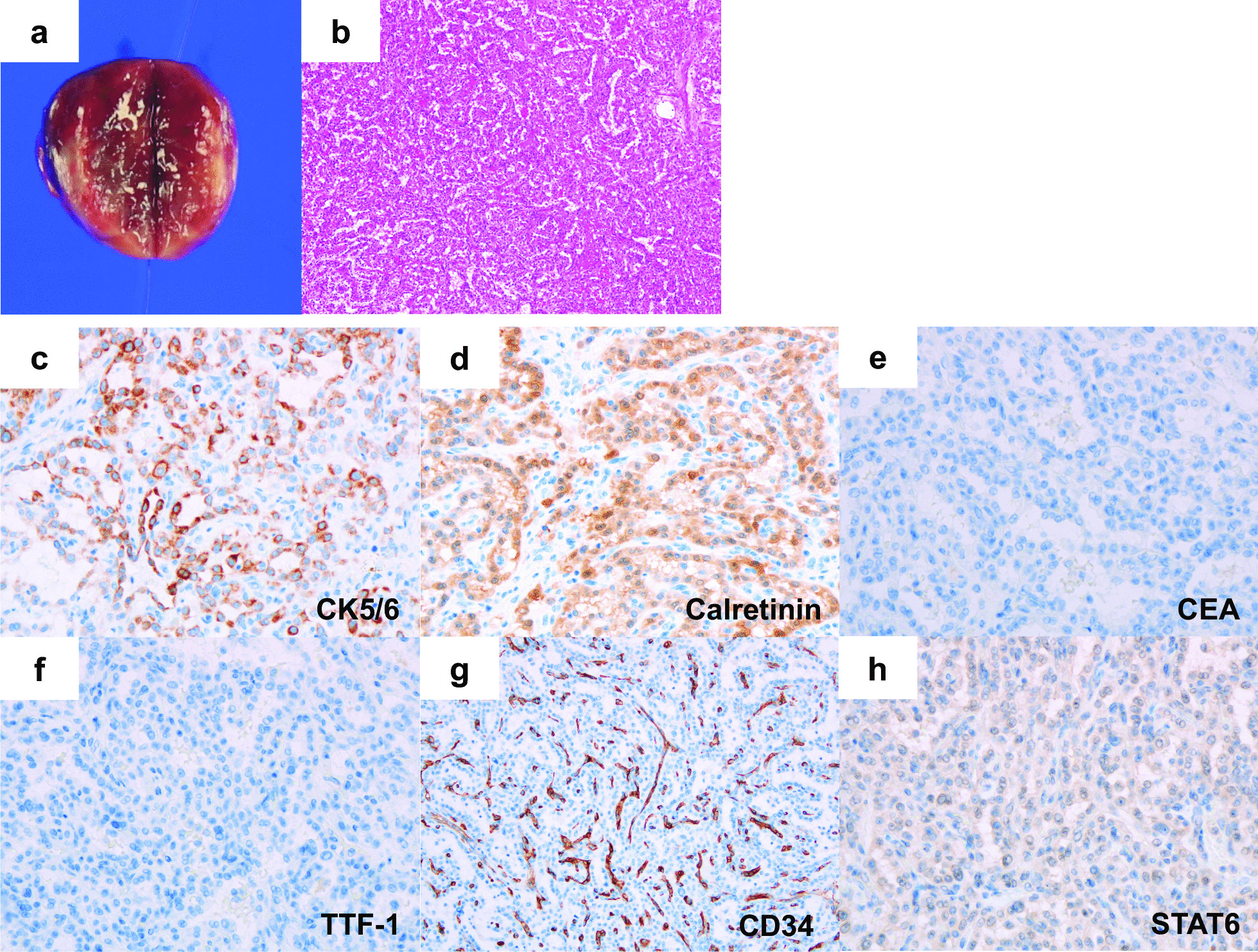
Macroscopic and microscopic findings of the resected tumor. **a** Macroscopic findings revealed a 28 × 20 × 11 mm^3^ brown–red tumor with a smooth cut surface. **b** Microscopic findings revealed papillary architecture with focal small aggregates of mesothelial cells. **c** Tumor cells were positive for cytokeratin 5/6. **d** Tumor cells were partially positive for calretinin. **e** Tumor cells were negative for carcinoembryonic antigen. **f** Tumor cells were negative for thyroid transcription factor-1. **g** Tumor cells were negative for cluster of differentiation 34. **h** Tumor cells were negative for signal transducer and activator of transcription 6

A 61-year-old Japanese woman underwent resection of a malignant peripheral nerve sheath tumor of the hand when she was aged 43 years and was followed up by radiological examination. Chest radiography revealed a mass lesion in the left upper lung 18 years later. She was a current smoker but had no history of asbestos exposure, and presented no specific clinical symptoms. As a result of a detailed examination, she was diagnosed with clinical stage I lung adenocarcinoma. Simultaneously, contrast-enhanced computed tomography (CT) detected a 20-mm enhancing nodule with slow growth on the right diaphragm (Fig. 1a). 18-Fluoro-2-deoxyglucose positron emission tomography revealed that the maximum standard uptake value of the nodule was 3.5 (Fig. 1b). Ultrasonography (US) revealed a low-echoic lesion, and early enhancement was observed on Sonazoid-enhanced US (Fig. 1c, d). These results indicated that the lesion was a hypervascular tumor of borderline malignancy, such as solitary fibrous tumor (SFT). After a left upper lobectomy for lung adenocarcinoma, the patient was referred to our department for surgical resection of the peritoneal tumor. Laboratory data at the time of presentation were as follows: white blood cell count, 7240 cells/μL; hemoglobin level, 14.2 g/dL; platelet count, 10.4 × 10^4^ cells/μL; aspartate transaminase, 22 IU/L; alanine aminotransferase, 24 IU/L; total bilirubin, 0.57 mg/dL; albumin, 4.1 g/dL; creatinine, 0.58 mg/dL. Serum tumor markers, such as proteins induced by vitamin K absence or antagonist II and alpha-fetoprotein, were within the normal range (25 μg/mL and 2.1 ng/mL, respectively). Laparoscopic tumor resection was performed. Intraoperative findings are shown in Fig. 2. A thin pedunculated tumor was found to originate from the peritoneal surface of the right diaphragm. The tumor was compressing liver segment 8 but without apparent invasion. Well-developed capillary vessels were observed around the tumor. The pedicle of the tumor was clipped at its origin and divided, and a tumorectomy was completed. Gross examination showed a 28 × 20 x 11 mm^3^ brown–red tumor with a smooth cut surface (Fig. 3a). Histopathological examination revealed papillary architecture with focal small aggregates of mesothelial cells (Fig. 3b). Glandular lumen formation, indicative of an adenomatoid pattern, was partially observed. Immunohistochemical analysis showed that the tumor cells were positive for cytokeratin 5/6 (CK 5/6) and calretinin, and negative for carcinoembryonic antigen (CEA), thyroid transcription factor-1 (TTF-1), cluster of differentiation 34 (CD34), and signal transducer and activator of transcription 6 (STAT6) (Fig. 3c–h). In addition, hot-spot mutations in *TNF receptor associated factor 7* (*TRAF7*) were not detected by Sanger sequencing, and the tumor cells displayed negative immunostaining for L1 cell adhesion molecule. Fluorescence in situ hybridization (FISH) showed no homozygous deletion of *9p21* or hemizygous deletion of *NF2* (data not shown). Based on these results, the patient was diagnosed with localized adenomatoid mesothelioma. The postoperative course was uneventful, and the patient was discharged on the fourth postoperative day. She remains alive and is being monitored in an outpatient setting. No recurrence was noted 6 months after surgery.

## Discussion and conclusions

Malignant mesothelioma is a highly aggressive tumor arising from mesothelial cells lining the pleura, peritoneum, and pericardium. The prognosis differs among histological subtypes, and epithelioid mesothelioma contains several morphological subtypes that also have some impact on prognosis [4, 5]. Adenomatoid mesothelioma is usually classified as an architectural subtype of epithelioid mesothelioma. It can histologically mimic adenomatoid tumors and is sometimes difficult to distinguish from others [3]. We report herein a case of peritoneal adenomatoid mesothelioma originating in the diaphragm. There are several reports of pleural adenomatoid mesothelioma, but adenomatoid mesothelioma of peritoneal origin is extremely rare. To the best of our knowledge, this is the first report of peritoneal adenomatoid mesothelioma localized to the diaphragm.

In the present patient, we made the final pathological diagnosis of localized adenomatoid mesothelioma based on histochemical and immunohistochemical analyses and genetic profiling. Based on the tumor growth rate and enhancement pattern on contrast-enhanced CT, we first suspected an SFT. However, immunohistochemical staining was negative for CD34 and STAT6, ruling out the possibility of SFTs. Metastasis of lung adenocarcinoma was also ruled out based on the immunohistochemical staining patterns for positive mesothelial and negative epithelial markers (positive for CK5/6 and calretinin and negative for CEA and TTF-1). The tumor was thought to be an adenomatoid tumor or adenomatoid mesothelioma. Several reports indicate that adenomatoid tumors are defined by *TRAF7* mutation; therefore, we examined *TRAF7* hot-spot mutations, including codons 519, 521, 538, 561, and 577, using Sanger sequencing [6, 7]. *TRAF7* mutations were not detected. Homozygous deletion of* 9p21* and hemizygous deletion of* NF2*, which are characteristics of pleural mesothelioma, was also not detected by FISH. However, the frequency of these genetic alterations in peritoneal mesothelioma is reported to be less than that of pleural mesothelioma, which led to the final diagnosis of adenomatoid mesothelioma [8]. In addition to a pathological examination, a genetic approach may be necessary and useful for distinguishing adenomatoid mesothelioma from adenomatoid tumors.

We conducted a literature search of the PubMed database to understand the current status of adenomatoid mesothelioma in the abdominal cavity. We came across six cases, including our case, that reported an “adenomatoid mesothelioma” (Table 1) [9–13]. There was one man and five women, and none had a history of asbestos exposure. The sites of tumor origin included the uterus (*n* = 1), testis (*n* = 1), liver (*n* = 1), and peritoneum (*n* = 4), and multiple tumors were observed in patient 5. Recurrence was not observed in five out of six patients, but Mori* et al*. reported a recurrent case after curative resection (patient 5) [13]. We should also conduct a careful follow-up with our patient, although the optimal follow-up strategy has not been well established. Through a literature search, we found that there were patients in whom it was difficult to distinguish between adenomatoid tumors and adenomatoid mesothelioma. For instance, several reports mentioned adenomatoid mesothelioma as a “benign tumor.” However, considering that adenomatoid mesothelioma is categorized as a subtype of epithelial mesothelioma in the latest World Health Organization classification, it should be treated as a malignant tumor. In addition, there are several reports of adenomatoid tumors invading the surrounding organs. Adenomatoid tumors are classified as benign; therefore, these patients may need to be recategorized according to the current classification. It is necessary to clearly define adenomatoid mesothelioma not only from the viewpoint of morphology but also from the viewpoint of mutational status, such as *TRAF7* mutation and immunostaining findings, which would enable us to examine the clinical characteristics of adenomatoid mesothelioma.

**Table 1:** Reported cases of adenomatoid mesothelioma in the abdominal cavity

Case	Study	Age	Sex	Asbestos	Tumor origin	Note	Diameter	Clinical symptoms	Surgery	Pathological diagnosis	Recurrence	Later course
1	Bisset et al. 1988	45	F	–	Uterus	Approximately 10 cm, No recurrence for 3 years	10 cm	Heavy, frequent menstrual bleeding	Hysterectomy	Giant cystic adenomatoid tumor (mesothelioma)	No, 3 years	Remains well 3 years later
2	Lins et al. 2009	25	F	–	Peritoneum	Approximately 10 cm, No recurrence for 7 years	10.8 cm	Pelvic pain	Tumorectomy	Adenomatoid mesothelioma	No, 7 years	Free of the disease for 7 years
3	Okuda et al. 2014	61	F	–	Peritoneum	Invasion to ovary	UK	None	Laparoscopic right salpingo-oophorectomy and adhesiotomy	Adenomatoid-like mesothelioma	UK	Two months later, the patient underwent laparoscopic segmental resection of the sigmoid colon, with histological analysis identifying an adenomatoid-like tumor. No information about later course
4	Yang et al. 2014	68	M	–	Testis	Invasion to tunica vaginalis and spermatic cord	6.8 cm	Swelling and slightly painful in the right scrotum	Radical orchiectomy	Mesothelioma with prominent adenomatoid features	No	The clinical follow-up was done at 3 months and 6 months after the surgery, respectively. No recurrence or metastasis of the tumor was observed
5	Mori et al. 2020	60	F	–	Lliver surface, pelvic cavity and anterior peritoneum	Invasion to blood vessel, multiple recurrences (peritoneum, lung). Alive at 7 years after surgery	UK	None	Laparoscopic tumor resection	Adenomatoid (microcystic) mesothelioma	Yes	Relapsed 4 years later and metastasized to the lung, but the patient remains alive 7 years after the first tumor resection surgery, while receiving chemotherapy.
6	Our case	61	F		Peritoneum (diaphragm)	No invasion	2.8 cm	None	Laparoscopic tumor resection	Adenomatoid mesothelioma	No, 6 months	

*UK* unknown

In conclusion, we encountered a case of adenomatoid mesothelioma of peritoneal origin. It is important to accumulate and share such experiences, which may lead to better characterization of this disease.

## Data Availability

Not applicable.

## Abbreviations

CT: Computed tomography
US: Ultrasonography
SFT: Solitary fibrous tumor
FISH: Fluorescence in situ hybridization
CEA: Carcinoembryonic antigen
TTF-1: Thyroid transcription factor-1
CD34: Cluster of differentiation 34
STAT6: Signal transducer and activator of transcription 6
*TRAF7*: *TNF receptor associated factor 7*
CK 5/6: Cytokeratin 5/6
